# Monosodium urate crystal deposition associated with the progress of radiographic grade at the sacroiliac joint in axial SpA: a dual-energy CT study

**DOI:** 10.1186/s13075-017-1286-0

**Published:** 2017-05-02

**Authors:** Junqing Zhu, Aiwu Li, Ertao Jia, Yi Zhou, Juan Xu, Shixian Chen, Yinger Huang, Xiang Xiao, Juan Li

**Affiliations:** 1 Department of Rheumatology, Nanfang Hospital, Southern Medical University, Guangzhou, Guangdong 510515 China; 2 Department of Internal Medicine of Traditional Chinese Medicine, College of Traditional Chinese Medicine, Southern Medical University, Guangzhou, Guangdong 510510 China; 3 Department of Obstetrics, Guangdong Women and Children Hospital, Guangzhou, Guangdong 511400 China; 4 Department of Radiology, Nanfang Hospital, Southern Medical University, Guangzhou, Guangdong 510515 China

**Keywords:** Axial Spondyloarthritis (AxSpA), Ankylosing spondylitis (AS), Dual-energy computed tomography (DECT), Monosodium urate (MSU) crystal, Sacroiliac joint

## Abstract

**Background:**

Previous studies have revealed that ankylosing spondylitis (AS), as the progenitor of axial spondyloarthritis (AxSpA), has been characterized by the insidiously progressive nature of sacroiliitis and spondylitis. Dual-energy computed tomography (DECT) has recently been used to analyse the deposition of monosodium urate (MSU) crystals with higher sensitivity and specificity. However, it remains unclear whether the existence of the MSU crystal deposition detected by DECT at the sacroiliac joint in patients with AxSpA also is associated with the existing structural damage. Here, we performed this study to show the DECT MSU crystal deposits in AxSpA patients without coexisting gout and to ascertain the relationship between the MSU crystal deposition and the structural joint damage of sacroiliac joints.

**Methods:**

One hundred and eighty-six AxSpA patients without coexisting gout were recruited. The plain radiographs of the sacroiliac joint were obtained, along with the DECT scans at the pelvis and the clinical variables. All statistics based on the left or right sacroiliac joint damage grading (0–4) were calculated independently. Bivariate analysis and ordinal logistic regression was performed between the clinical features and radiographic grades at the sacroiliac joint.

**Results:**

At the pelvis, large quantities of MSU crystal deposition were found in patients with AxSpA. The average MSU crystal volume at the left sacroiliac joint, the right sacroiliac joint, and the pelvis were 0.902 ± 1.345, 1.074 ± 1.878, and 5.272 ± 9.044 cm^3^, values which were correlated with serum uric acid concentrations (*r* = 0.727, 0.740, 0.896; *p* < 0.001). In bivariate analysis, wide clinical variables were associated with the changes in sacroiliac joint damage. Further, the AxSpA duration, BASFI score, and the volume of MSU crystal at both sides of sacroiliac joint were associated with the progress of radiographic grade at the sacroiliac joints in the ordinal logistic models (left AOR = 1.180, 3.800, 1.920; right AOR = 1.190, 3.034, 1.418; *p* < 0.01).

**Conclusions:**

Large quantities of MSU crystal deposition detected by DECT were found at the pelvis in AxSpA patients without coexisting gout. In addition to AxSpA duration and BASFI score, the MSU crystal deposition at the sacroiliac joint is associated with the progress of radiographic grade at sacroiliac joints in those patients.

## Background

The term spondyloarthritis (SpA) encompasses a group of diseases including axial SpA (AxSpA) and peripheral SpA, classifications which have been proposed by the Assessment of SpondyloArthritis International Society (ASAS) [1, 2]. It is estimated that SpA, with an incidence between 0.5 and 10.6 per 100,000 people per year, affects approximately 0.4–1.9% of the population across the globe [3]. In addition to what is now known as nonradiographic AxSpA (nr-AxSpA), ankylosing spondylitis (AS) has been characterized by the insidiously progressive nature of sacroiliitis and spondylitis, which leads to a significant reduction in quality of life and an increased mortality rate [4]. Although the aetiology and pathogenesis of AxSpA has not yet been fully elucidated, the current view is that it involves both inflammatory erosive osteopenia and unusual bony overgrowth [5, 6]. Precisely because the primal mechanism in autoimmune disorders is the loss of tolerance to self-proteins by environment-gene interactions, the coexistence of different rheumatic diseases is common. For example, AS grouped under the term AxSpA occurs in patients with rheumatoid arthritis (RA) [7, 8], gouty arthritis [9], multiple sclerosis [10, 11], and systemic lupus erythematosus [12].

There are few epidemiological publications regarding the coexistence of AxSpA and gout, but the study has revealed that AS concurrent with gout is more common than previously believed [9]. Although AxSpA and gout are two distinct rheumatic diseases, they share a few clinical characteristics, including inflammatory joint pain and excellent response to non-steroidal anti-inflammatory drugs (NSAIDs). AxSpA usually leads to structural damage and functional limitation, exemplified in patients with AS [13]. At the same time, structural joint damage is also frequently observed in patients with advanced gout as a result of monosodium urate (MSU) crystal deposition [14–16]. Therefore, in patients with coexisting AS and gout, it is hard to discriminate whether the inflammatory joint pain and structural joint damage are due to AS disease activity, the MSU crystal deposition, or both.

Dual-energy computed tomography (DECT) has been used to analyse the deposition of MSU crystal with high sensitivity (100%) and specificity (89%) [17]. The sensitivity and specificity of DECT for gout were only 0.87 and 0.84 [18], because the deposition of MSU crystal is a necessary but not a sufficient condition. Besides, DECT MSU crystal deposition was observed in both multiple joints and soft tissues in patients with asymptomatic hyperuricaemia, and was associated with increasingly severe coronary calcification [19, 20]. In addition, our study found a large quantity of MSU crystal deposition with DECT in AxSpA patients without coexisting gout, according to the 1977 American Rheumatism Association classification criteria [21]. However, whether the existence of MSU crystal deposition in those patients has contributed to the structural joint damage is still unclear.

The purpose of our study was to show the DECT MSU crystal deposits at the pelvis in AxSpA patients without coexisting gout, and further to analyse whether the MSU crystal deposition at the sacroiliac joint is associated with the structural joint damage of the sacroiliac joint in those patients.

## Methods

One hundred and eighty-six patients with AxSpA were recruited from rheumatology clinics at Nanfang Hospital in China during the period of October 2012 to July 2015. All participants had AxSpA described by the ASAS classification criteria [1, 2] and had not been diagnosed with gout, according to the 1977 American Rheumatism Association classification criteria [21].

The plain radiographs of the sacroiliac joint, a DECT scan of pelvis, and the clinical variables were obtained at a follow-up appointment. In brief, each sacroiliac joint on a plain radiograph was graded on a scale of 0–4 according to the modified New York criteria [22]. DECT scans were performed on a dual-source X-ray tube 128 detector row scanner (Somatom Definition Flash, Siemens Healthcare, Erlangen, Germany). All scans were performed using the same image protocol: acquisition at 128 mm × 0.6 mm and pitch of 0.7. The two X-ray tubes are operated simultaneously at 80 kV and 140 kV. The image reconstructions were done using proprietary software (Siemens Multimodality Workplace, Software version MMWP Syngo CT 2010A, Siemens Healthcare, Erlangen, Germany), with a 512 × 512 matrix, to 0.75-mm slices, with a 0.5-mm increment. The parameter ratio for urate was set at 1.28. Two readers, blinded to the clinical variables and plain radiographic scores, evaluated the DECT scans for the presence and volumes of MSU crystal, independently. MSU crystal was considered present at each site only if reported by both readers and the average volume of MSU crystal was calculated [23].

The clinical variables, including age, gender, AxSpA disease duration, HLA-B27 positivity, erythrocyte sedimentation rate (ESR, mm/h), C-reactive protein (CRP, mg/l), total back pain (10-cm visual analogue scale, VAS), patient’s global assessment (PGA) of disease activity (10-cm VAS), pain and swelling of peripheral arthritis (10-cm VAS), duration of morning stiffness (10-cm VAS), use of non-steroidal anti-inflammatory drugs (NSAIDs), use of disease-modifying anti-rheumatic drugs (DMARDs), use of biologic DMARDs, serum uric acid (μmol/L), MSU crystal positivity, and volume of MSU crystal (cm^3^) were all assessed. In addition, the Ankylosing Spondylitis Disease Activity Score (ASDAS) [24] and the grading for disease activity [25] were calculated as previous study. The Bath Ankylosing Spondylitis Functional Index (BASFI) scores were calculated according to the average scores of ten questions [26].

All statistics based on the left or right sacroiliac joint damage grading were calculated independently with IBM SPSS (Version 20.0, IBM Corp., Armonk, NY, USA). Measurement data were presented as mean ± standard deviation (mean ± SD) or with a 95% confidence interval (CI), while count data were presented as numbers (n). One-way analysis of variance (ANOVA) was used to evaluate the statistical differences among groups. The non-normal distribution measurement data were tested with the Kruskal-Wallis rank-sum test. To count data, Pearson’s chi-square (*x* ^*2*^) and Fisher’s exact test were used for the comparison. Interobserver reproducibility for the volume of MSU crystals by two readers was assessed by the intraclass correlation coefficient (ICC) and limits of agreement by Bland-Altman analysis [27]. The ICC values, 95%CI, and the *p* values were reported. Spearman’s correlation analysis was performed to evaluate the association between serum uric acid and the average volume of MSU crystal. Spearman’s rank correlation coefficient (r) and *p* values were reported. Bivariate analysis was performed between the clinical features and radiographic grades. The odds ratios (OR) and its 95%CI were reported. In addition, the ordinal logistic regression was used to identify the effect of each potential factor adjusted for others. Variables with *p* < 0.05 in bivariate analysis were included in the ordinal logistic models. The adjusted odds ratios (AOR) 95%CI were reported. A test of parallel lines was also performed to evaluate the appropriateness of the ordinal logistic model. A *p* < 0.05 was considered statistically significant. All *p* values were two-tailed.

## Results

### Clinical characteristics of the patients with AxSpA

The 186 patients’ clinical features are shown in Table 1. Patients were predominantly young males and were HLA-B27 positive. Mean disease duration was 4.3 years, mean ESR was 25.1 mm/h, and mean serum uric acid was 362.7 μmol/L. The median ASDAS scores and BASFI scores were 2.7 ± 1.1 and 4.3 ± 1.1. Seventeen and fifty-seven patients had never used NSAIDs or biologic DMARDs. Seventeen patients had used DMARDs for less than 3 months. Large quantities of MSU crystal deposition detected by DECT were found at the pelvis in AxSpA patients without coexisting gout. The positive rates of MSU crystal at the sacroiliac joint, hip joint, and pubic symphysis were 111 (29.8%), 75 (40.3%), and 63 (33.9%). The average volumes of MSU crystal at the sacroiliac joint and pelvis were 0.29 ± 0.99 and 4.37 ± 8.46 cm^3^.

**Table 1:** Characteristics of the patients with AxSpA included in the study

	Total	Based on the left sacroiliac joint damage					*p* value	Based on the right sacroiliac joint damage					*p* value
		0 grade	1 grade	2 grade	3 grade	4 grade		0 grade	1 grade	2 grade	3 grade	4 grade	
Number of cases (n)	186	4	8	73	72	29	NA	4	10	74	72	26	NA
Age (years)	26.4 ± 8.7	25.3 ± 5.4	29.4 ± 10.7	24.9 ± 8.7	27. 6 ± 8.3	26.5 ± 9.2	0.138	25.3 ± 5.4	25.7 ± 9.4	25.0 ± 8.6	27.9 ± 8.5	26.7 ± 9.4	0.133
Male/female (n)	143/43	3/1	6/2	55/18	54/18	25/4	0.763	3/1	7/3	57/17	53/19	23/3	0.564
AxSpA duration (years)	4.3 ± 4.0	0.8 ± 0.3	4.4 ± 2.5	3.1 ± 3.1	4.8 ± 3.7	6.9 ± 5.6	<0.001	0.8 ± 0.3	4.5 ± 2.9	2.8 ± 2.2	5.1 ± 4.3	7.0 ± 5.5	<0.001
HLA-B27 (+/-) (n)	164/22	3/1	6/2	68/8	65/7	25/4	0.725	3/1	9/1	64/10	66/6	22/4	0.741
ESR (mm/h)	25.1 ± 22.1	9.5 ± 10.6	10.1 ± 8.9	20.2 ± 16.5	29.1 ± 24.8	33.7 ± 25.7	0.002	9.5 ± 10.6	23.8 ± 20.1	19.2 ± 17.2	28.3 ± 23.9	35.8 ± 26.3	0.007
CRP (mg/l)	14.3 ± 17.5	1.0 ± 0.5	3.1 ± 2.4	9.2 ± 13.1	16. 6 ± 17.2	26.0 ± 23.4	<0.001	1.0 ± 0.5	9.4 ± 15.6	9.9 ± 14.3	15.1 ± 15.9	28.1 ± 23.8	<0.001
Total back pain^#^	4.9 ± 2.8	1.3 ± 0.5	1.9 ± 1.4	4.5 ± 2.6	5.4 ± 2.6	6.3 ± 2.8	<0.001	1.3 ± 0.5	3.6 ± 3.2	4.5 ± 2.6	5.2 ± 2.6	6.6 ± 2.8	<0.001
PGA of disease activity^#^	5.6 ± 3.0	2.5 ± 1.9	2.5 ± 1.8	5.3 ± 2.8	5.9 ± 2.9	7.0 ± 2.9	<0.001	2.5 ± 1.9	4.4 ± 3.7	5.1 ± 2.8	5.9 ± 2.7	7.3 ± 3.0	0.002
Pain and swelling of peripheral arthritis^#^	0.2 ± 0.5	NA	0.3 ± 0.4	0.2 ± 0.4	0.3 ± 0.5	0.4 ± 0.6	0.249	NA	0.2 ± 0.4	0.2 ± 0.4	0.3 ± 0.5	0.4 ± 0.6	0.227
Duration of morning stiffness^#^	5.4 ± 2.8	2.8 ± 1.0	2.0 ± 1.3	5.1 ± 2.7	5.8 ± 2.6	6.4 ± 2.9	<0.001	2.8 ± 1.0	4.0 ± 3.6	4.9 ± 2.6	5.8 ± 2.5	6.7 ± 2.9	0.003
ASDAS (scores)	2.7 ± 1.1	1.0 ± 0.2	1.4 ± 0.4	2.4 ± 1.3	3.0 ± 1.3	3.5 ± 1.5	<0.001	1.0 ± 0.2	2.1 ± 1.6	2.4 ± 1.3	2.9 ± 1.3	3.6 ± 1.6	<0.001
BASFI (scores)	4.3 ± 1.1	1.9 ± 0.8	2.5 ± 0.9	4.0 ± 1.0	4.6 ± 0.7	4.9 ± 0.8	<0.001	1.9 ± 0.8	3.1 ± 1.5	4.1 ± 1.0	4.5 ± 0.8	4.9 ± 0.8	<0.001
Use of NSAIDs (ever/never) (n)	169/17	0/4	8/0	68/5	67/5	26/3	<0.001	0/4	10/0	70/4	66/6	23/3	<0.001
Use of DMARDs (≥3/<3 months) (n)	169/17	1/3	6/2	69/4	68/4	25/4	0.005	1/3	8/2	70/4	67/5	23/3	0.010
Ever use of biologic DMARDs (n)													
> 12 months	28	0	4	18	4	2	<0.001	0	5	15	6	2	<0.001
≤ 12 months	42	1	3	21	15	2		1	3	24	12	2	
≤ 6 months	59	2	1	16	30	10		2	0	18	32	7	
Never	57	1	0	18	23	15		1	2	17	22	15	
Serum uric acid (μmol/L)	362.7 ± 107.9	284.0 ± 95.3	345.1 ± 77.0	361.9 ± 105.5	366.0 ± 117.7	372.2 ± 97.8	0.593	284.0 ± 95.3	325.6 ± 103.5	362.3 ± 101.9	372.4 ± 118.8	363.3 ± 94.6	0.499
MSU crystallization (+/-) (n)													
Sacroiliac joint	111/261^$^	0/4	0/8	14/59	29/43	14/15	<0.001	0/4	1/9	13/61	28/44	12/44	0.002
Hip joint	75/111	1/3	4/4	33/40	28/44	9/20	0.632	1/3	6/4	32/42	29/43	7/19	0.369
Pubic symphysis	63/123	0/4	3/5	25/48	28/44	7/22	0.244	0/4	4/6	25/49	29/43	5/21	0.112
Volume of MSU crystallization (cm^3^)													
Sacroiliac joint	0.29 ± 0.99^$^	NA	NA	0.10 ± 0.30	0.31 ± 0.69	0.75 ± 1.72	0.001	NA	0.01 ± 0.01	0.10 ± 0.32	0.41 ± 1.19	0.83 ± 2.11	0.005
Total volume at pelvis	4.37 ± 8.46	0.02 ± 0.03	0.63 ± 0.99	4.36 ± 8.01	4.92 ± 10.03	4.62 ± 6.72	0.077	0.02 ± 0.03	2.15 ± 4.16	4.00 ± 7.73	5.51 ± 10.37	3.78 ± 5.91	0.118

Values are given as the numbers or the mean ± standard deviation (Mean±SD);

*AxSpA* axial spondyloarthritis, *n* numbers, *+/-* positive/negative, *ESR* erythrocyte sedimentation rate, *CRP* C-reactive protein, *PGA* patient’s global assessment, *ASDAS* Ankylosing Spondylitis Disease Activity Score, *BASFI* Bath Ankylosing Spondylitis Functional Index, *NSAIDs* non-steroidal anti-inflammatory drugs, *DMARDs* disease-modifying anti-rheumatic drugs, *MSU* monosodium urate, *NA* not available

^#^All assessed on a visual analogue scale (0–10 cm)

^$^The sum of the data from the left and right sacroiliac joint

The statistically significant results were found among the radiographic grade group at the left or right sacroiliac joints for disease duration, ESR, CRP, total back pain, PGA of disease activity, duration of morning stiffness, ASDAS scores, BASFI scores, use of NSAIDs/DMARDs/biologic DMARDs, as well as the presence and volume of MSU crystal on the left and right sacroiliac joints (*p* < 0.05) (Table 1).

### Reproducibility of DECT MSU crystal volume measurement and correlation between the average MSU crystal volume and serum uric acid

**Fig. 1:**
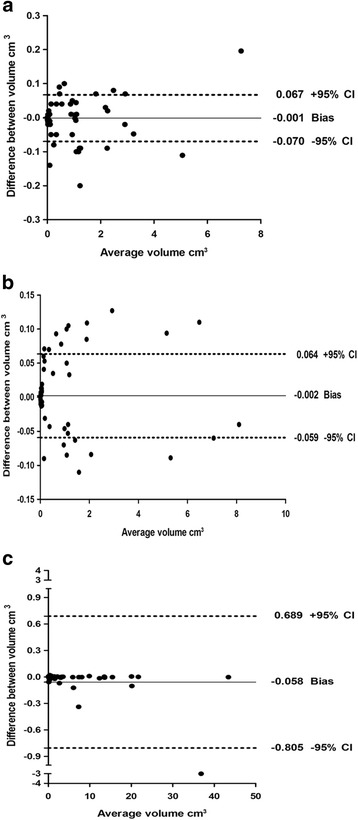
Bland-Altman plots for interobserver reproducibility analysis. **a** The dual-energy computed tomography (DECT) monosodium urate (MSU) crystal volume at the *left* sacroiliac joint. **b** The DECT MSU crystal volume at the *right* sacroiliac joint. **c** The DECT MSU crystal volume at the pelvis. *Solid line* shows bias and *dashed lines* show the 95% limits of agreement

Of the patients who showed the presence of MSU crystal deposition, 57 showed deposition at the left sacroiliac joint, 54 at the right sacroiliac joint, and 154 at the pelvis. The interobserver reproducibility analysis is shown in Table 2. ICCs for MSU crystal volume measurements at the sacroiliac joint and pelvis were all greater than 0.99 (*p* < 0.001). Bland-Altman plots illustrating the interobserver limits of agreement for MSU crystal volume measurements are shown in Fig. 1. The average MSU crystal volume measurements at the left sacroiliac joint, the right sacroiliac joint, and the pelvis were 0.902 ± 1.345, 1.074 ± 1.878, and 5.272 ± 9.044 cm^3^. For further analysis, the serum uric acid concentration was correlated with the volumes of MSU crystal at the left sacroiliac joint (*r* = 0.727, *p* < 0.001), the right sacroiliac joint (*r* = 0.740, *p* < 0.001), and the pelvis (*r* = 0.896, *p* < 0.001) (Table 2).

**Table 2:** The interreader reproducibility analysis of MSU crystal volume and its correlation with the serum uric acid

Joint	Reader	Volume of MSU crystallization (cm^3^)		Intraclass correlation coefficient			Correlation with serum uric acid^#^	
		Mean ± SD	95% CI	*ICC*	95% CI	*p*	*r*	*p*
Left sacroiliac joint (n = 57)	Reader 1	0.900 ± 1.351	0.541–1.258	0.999	0.998-0.999	<0.001	NA	NA
	Reader 2	0.904 ± 1.340	0.549–1.260				NA	NA
	Average	0.902 ± 1.345	0.545–1.259	NA	NA	NA	0.727	<0.001
Right sacroiliac joint (n = 54)	Reader 1	1.078 ± 1.878	0.578–1.619	1.000	0.999-1.000	<0.001	NA	NA
	Reader 2	1.071 ± 1.878	0.5700–1.612				NA	NA
	Average	1.074 ± 1.878	0.574–1.616	NA	NA	NA	0.740	<0.001
Pelvis (n = 154)	Reader 1	5.237 ± 8.942	3.813–6.660	0.999	0.999-0.999	<0.001	NA	NA
	Reader 2	5.307 ± 9.150	3.850–6.764				NA	NA
	Average	5.272 ± 9.044	3.832–6.712	NA	NA	NA	0.896	<0.001

Values are given as the mean ± standard deviation (mean ± SD)

*MSU* monosodium urate, *ICC* intraclass correlation coefficient values, *95% CI* 95% confidence interval, r, Spearman rank correlation coefficient, *NA* not available

^#^Spearman correlation analysis between serum uric acid and the average volume of MSU crystals

### Comparison of MSU crystal deposition and serum uric acid in patients grouped by ASDAS scores

According to the ASDAS scores, all patients were divided into four groups (Table 3). The presence of MSU crystal deposition at the left and right sacroiliac joints showed statistical differences among groups (*x* ^*2*^ = 11.451, 43.684; *p* = 0.010, <0.001). However, there was no statistical difference among groups at the hip joint and pubic symphysis for the presence of MSU crystal deposition (*x* ^*2*^ = 0.676, 0.549; *p* = 0.879, 0.908). Statistical differences were found in regards to the volumes of MSU crystal at the left sacroiliac joint (*Z* = 9.198, *p* = 0.027), the right sacroiliac joint (*Z* = 34.607, *p* < 0.001), and the pelvis (*Z* = 10.517; *p* = 0.015). In addition the ANOVA tests revealed that the serum uric acid concentration was statistically different among groups (*F* = 6.322, *p* < 0.001).

**Table 3:** Comparison of MSU crystal deposits at different regions and serum uric acid in patients grouped by ASDAS scores

	ASDAS scores				*x* ^*2*^ */Z/F*	*p*
	ASDAS < 1.3 (n = 38)	1.3 ≤ ASDAS < 2.1 (n = 38)	2.1 ≤ ASDAS ≤ 3.5 (n = 50)	ASDAS > 3.5 (n = 60)		
MSU crystallization (+/-) (n)^#^						
Left sacroiliac joint	8/30	6/32	16/34	27/33	11.451	0.010
Right sacroiliac joint	3/35	4/34	11/39	36/24	43.684	<0.001
Hip joint	15/23	16/22	22/28	22/38	0.676	0.879
Pubic symphysis	11/27	13/25	18/32	21/39	0.549	0.908
Volume of MSU crystallization (cm^3^)^$^						
Left sacroiliac joint	0.21 ± 0.62	0.14 ± 0.47	0.49 ± 1.34	0.23 ± 0.57	9.198	0.027
Right sacroiliac joint	0.04 ± 0.18	0.13 ± 0.52	0.35 ± 1.09	0.57 ± 1.62	34.607	<0.001
Total volume at pelvis	2.41 ± 6.51	2.69 ± 5.04	7.40 ± 11.49	4.13 ± 7.77	10.517	0.015
Serum uric acid (μmol/L)&	321.0 ± 100.4	326.2 ± 104.2	397.9 ± 109.4	382.9 ± 99.8	6.322	<0.001

Values are given as the numbers or the mean ± standard deviation (Mean±SD)

*ASDAS* Ankylosing Spondylitis Disease Activity Score, *MSU* monosodium urate

^#^Pearson chi-square (*x* ^*2*^) test

^$^Kruskal-Wallis rank-sum test

&One-way analysis of variance (ANOVA)

### Unadjusted associations of clinical features with the grade of sacroiliac joint damage

In bivariate analysis (Table 4), wide clinical variables were associated with the changes in sacroiliac joint damage based on the radiographic grade at the left sacroiliac joint. The OR scores were 1.189 (95%CI 1.103–1.283) for disease duration, 1.024 (95%CI 1.011–1.037) for ESR, 1.045 (95%CI 1.027–1.063) for CRP, 1.298 (95%CI 1.169–1.440) for total back pain, 1.214 (95%CI 1.104–1.335) for PGA of disease activity, 1.849 (95%CI 1.051–3.254) for pain and swelling of peripheral arthritis, 1.242 (95%CI 1.122–1.376) for duration of morning stiffness, 1.716 (95%CI 1.397–2.107) for ASDAS scores, 3.188 (95%CI 2.330–4.364) for BASFI scores, 0.143 (95%CI 0.057–0.357) for use of biologic DMARDs more than 12 months, and 0.274 (95%CI 0.127–0.592) for use of biologic DMARDs more than 6 months. Positive associations were also observed between changes in left sacroiliac joint damage and the presence and volume of MSU crystal on the left sacroiliac joint (OR = 3.368, 1.990; 95%CI 1.841–6.160, 1.308–3.028). However, the changes in left sacroiliac joint damage were not significantly associated with age, gender (male), HLA-B27-positive status, any use of NSAIDs, use of DMARDs more than 3 months, serum uric acid, MSU crystal on the hip joint and pubic symphysis, and volume of MSU crystal on the pelvis. The same trend results were obtained in bivariate analysis for the changes in sacroiliac joint damage at the right sacroiliac joint.

**Table 4:** Bivariate analysis between the grade of sacroiliac joint damage and clinical features^#^

Characteristics	Based on the left sacroiliac joint damage			Based on the right sacroiliac joint damage		
	Unadjusted *OR*	*95% CI*	*p* value	Unadjusted *OR*	*95% CI*	*p* value
Age, per year	1.016	0.985–1.047	0.324	1.025	0.994–1.057	0.121
Male, vs female	1.303	0.692–2.454	0.412	1.281	0.680–-2.412	0.444
AxSpA duration, per year	1.189	1.103–1.283	<0.001	1.195	1.108–1.289	<0.001
HLA-B27 positive, vs negative	1.248	0.546–2.850	0.599	1.197	0.524–2.732	0.670
ESR, per mm/h	1.024	1.011–1.037	<0.001	1.023	1.010–1.036	<0.001
CRP, per mg/l	1.045	1.027–1.063	<0.001	1.041	1.024–1.059	<0.001
Total back pain, per score	1.298	1.169–1.440	<0.001	1.263	1.139–1.399	<0.001
PGA of disease activity, per score	1.214	1.104–1.335	<0.001	1.221	1.110–1.343	<0.001
Pain and swelling of peripheral arthritis, per score	1.849	1.051–3.254	0.033	1.950	1.105–3.441	0.021
Duration of morning stiffness, per score	1.242	1.122–1.376	<0.001	1.234	1.115–1.367	<0.001
ASDAS, per score	1.716	1.397–2.107	<0.001	1.671	1.362–2.048	<0.001
BASFI, per score	3.188	2.330–4.364	<0.001	2.811	2.081–3.797	<0.001
Ever use of NSAIDs, vs never	1.893	0.740–4.842	0.183	1.372	0.543–3.471	0.504
Use of DMARDs ≥3 months, vs <3 months	1.922	0.751–4.922	0.173	1.967	0.769–5.034	0.158
Ever use of biologic DMARDs, vs never						
≥ 12 months	0.143	0.057–0.357	<0.001	0.182	0.074–0.444	<0.001
≤ 12 months	0.274	0.127–0.592	0.001	0.244	0.113–0.531	<0.001
≤ 6 months	0.795	0.404–1.567	0.508	0.757	0.383–1.493	0.421
Never	NA	NA	NA	NA	NA	NA
Serum uric acid, per μmol/L	1.001	0.999–1.004	0.358	1.001	0.999–1.004	0.276
MSU crystallization positive, vs negative						
Left sacroiliac joint	3.368	1.841–6.160	<0.001	NA	NA	NA
Right sacroiliac joint	NA	NA	NA	3.225	1.749–5.946	<0.001
Hip joint	0.715	0.415–1.232	0.227	0.674	0.390–1.163	0.156
Pubic symphysis	0.971	0.554–1.703	0.919	0.928	0.529–1.628	0.795
Volume of MSU crystallization, per cm^3^						
Left sacroiliac joint	1.990	1.308–3.028	0.001	NA	NA	NA
Right sacroiliac joint	NA	NA	NA	1.470	1.125–1.922	0.005
Total volume at pelvis	1.015	0.983–1.047	0.370	1.015	0.983–1.047	0.367

*AxSpA* axial spondyloarthritis, *ESR* erythrocyte sedimentation rate, *CRP* C-reactive protein, *PGA* patient’s global assessment, *ASDAS* Ankylosing Spondylitis Disease Activity Score, *BASFI* Bath Ankylosing Spondylitis Functional Index, *NSAIDs* non-steroidal anti-inflammatory drugs, *DMARDs* disease-modifying anti-rheumatic drugs, *MSU* monosodium urate, *OR* odds ratios, *95% CI* 95% confidence interval, *NA* not available

^#^Including clinical variables and the DECT scans results

### Adjusted associations of clinical features with the grade of sacroiliac joint damage

In the ordinal logistic models, variables with *p* < 0.05 in bivariate analysis were included (complex model 1 and 3, Table 5). For the radiographic grade at the left sacroiliac joint, the adjusted factors were AxSpA duration, total back pain, BASFI score, and the volume of MSU crystal at the left sacroiliac joint (AOR = 1.187, 1.428, 3.837, 2.018; 95%CI 1.089–1.294, 1.040–1.962, 2.263–6.506, 1.144–3.560; *p* < 0.001, *p* = 0.028, *p* < 0.001, *p* = 0.015). The same adjusted factors were obtained at the right sacroiliac joint, except the variable of total back pain. The AOR values were 1.188, 3.092, and 1.387 (95%CI 1.090–1.295, 1.896–5.044, 1.022–1.882; *p* < 0.001, *p* < 0.001, *p* = 0.036) for the variables of AxSpA duration, BASFI score, and the volume of MSU crystal at the right sacroiliac joint.

**Table 5:** The ordinal logistic regression analysis of clinical features # independently associated with the grade of sacroiliac joint damage

Characteristics	Based on the left sacroiliac joint damage							Based on the right sacroiliac joint damage				
	Complex model 1			Simplified model 2				Complex model 3		Simplified model 4		
	*AOR*	*95% CI*	*p* value	*AOR*	*95% CI*	*p* value	*AOR*	*95% CI*	*p* value	*AOR*	*95% CI*	*p* value
AxSpA duration, per year	1.187	1.089–1.294	0.000	1.180	1.086–1.283	0.000	1.188	1.090–1.295	0.000	1.190	1.096–1.293	0.000
ESR, per mm/h	1.003	0.981–1.025	0.794	1.003	0.982–1.025	0.755	1.001	0.981–1.022	0.905	1.003	0.982–1.024	0.781
CRP, per mg/l	1.038	0.999–1.079	0.058	1.018	0.991–1.046	0.194	1.025	0.987–1.065	0.196	1.008	0.981–1.036	0.576
Total back pain, per score	1.428	1.040–1.962	0.028	1.246	0.975–1.591	0.078	1.315	0.959–1.803	0.089	1.166	0.916–1.484	0.212
PGA of disease activity, per score	1.040	0.784–1.380	0.786	0.975	0.746–1.275	0.856	1.023	0.780–1.342	0.868	0.970	0.751–1.252	0.815
Pain and swelling of peripheral arthritis, per score	1.517	0.795–2.897	0.207	1.356	0.725–2.534	0.341	1.858	0.978–3.532	0.059	1.657	0.890–3.084	0.111
Duration of morning stiffness, per score	0.723	0.519–1.008	0.056	0.682	0.494–0.942	0.020	0.836	0.607–1.150	0.271	0.792	0.581–1.079	0.139
ASDAS, per score	0.492	0.179–1.354	0.170	NA	NA	NA	0.526	0.194–1.429	0.208	NA	NA	NA
BASFI, per score	3.837	2.263–6.506	0.000	3.800	2.250–6.417	0.000	3.092	1.896–5.044	0.000	3.034	1.870–4.922	0.000
Ever use of biologic DMARDs, vs never												
≥ 12 months	1.051	0.308–3.581	0.937	1.144	0.337–3.885	0.829	1.425	0.417–4.872	0.572	1.414	0.421–4.754	0.575
≤ 12 months	0.961	0.333–2.778	0.942	1.098	0.385–3.132	0.861	0.971	0.329–2.866	0.958	1.020	0.357–2.916	0.971
≤ 6 months	1.999	0.806–4.962	0.135	1.830	0.745–4.495	0.188	2.461	0.976–6.204	0.056	2.128	0.869–5.211	0.099
Never	NA	NA	NA	NA	NA	NA	NA	NA	NA	NA	NA	NA
MSU crystallization positive, vs negative												
Left sacroiliac joint	0.895	0.391–2.049	0.794	NA	NA	NA	NA	NA	NA	NA	NA	NA
Right sacroiliac joint	NA	NA	NA	NA	NA	NA	1.291	0.542–3.075	0.565	NA	NA	NA
Volume of MSU crystallization, per cm^3^												
Left sacroiliac joint	2.018	1.144–3.560	0.015	1.920	1.209–3.049	0.006	NA	NA	NA	NA	NA	NA
Right sacroiliac joint	NA	NA	NA	NA	NA	NA	1.387	1.022–1.882	0.036	1.418	1.075–1.870	0.014

Complex model 1 and 3, variables with *p* < 0.05 in bivariate analysis were included based on the radiographic grade at each sacroiliac joint; simplified model 2 and 4, the models with the repeated variables of the ASDAS score and MSU crystals deposits positive were excluded

*AxSpA* axial spondyloarthritis, *ESR* erythrocyte sedimentation rate, *CRP* C-reactive protein, *PGA* patient’s global assessment, *ASDAS* Ankylosing Spondylitis Disease Activity Score, *BASFI* Bath Ankylosing Spondylitis Functional Index, *DMARDs* disease-modifying anti-rheumatic drugs, *MSU* monosodium urate, *AOR* adjusted odds ratios, *95% CI* 95% confidence interval, *NA* not available

^#^Including clinical variables and the DECT scans results

In addition, the simplified models (simplified model 2 and 4, Table 5), which excluded the repeated variables of the ASDAS score and presence of MSU crystal deposition, were also performed. The AxSpA duration, BASFI score, and the volume of MSU crystal at the left sacroiliac joint (AOR = 1.180, 3.800, 1.920; 95%CI 1.086–1.283, 2.250–6.417, 1.209–3.049; *p* < 0.001, *p* < 0.001, *p* = 0.006) were associated with the progress of radiographic grade at the left sacroiliac joint after being adjusted for other potential factors, while the duration of morning stiffness was a protective factor (AOR = 0.682, 95%CI 0.494–0.942, *p* = 0.020). In the simplified ordinal logistic model at the right sacroiliac joint, AxSpA duration, BASFI score, and the volume of MSU crystal at the right sacroiliac joint were the main factors when others factors were adjusted (AOR = 1.190, 3.034, 1.418; 95%CI 1.096–1.293, 1.870–4.922, 1.075–1.870; *p* < 0.001, *p* < 0.001, *p* = 0.014).

The test for parallel lines was not significant in the complex model 1, simplified model 2, and simplified model 4 (*x* ^*2*^ = 34.904, 13.028, 40.234; *p* = 0.773, 1.000, 0.288), which suggests that the models are appropriate. However, the parameter estimation is less stable for complex model 2 (*x* ^*2*^ = 59.877, *p* = 0.036).

### The MSU crystal at the sacroiliac joint with radiographic sacroiliac joint damage in four separate patients with AxSpA

**Fig. 2:**
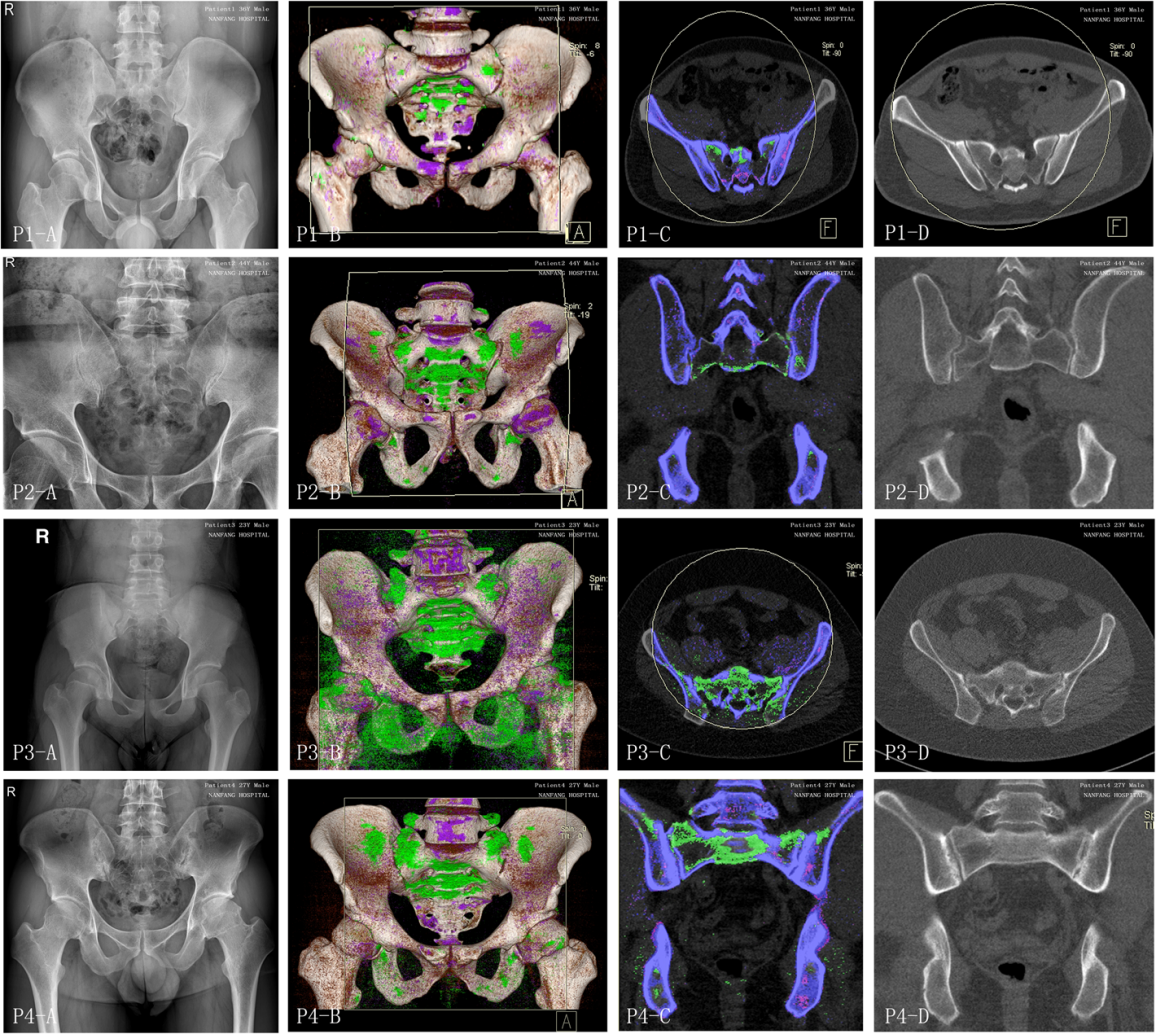
Four axial spondyloarthritis (AxSpA) patients with monosodium urate (MSU) crystal and radiographic structural damage at the sacroiliac joint. For each set of images, panel **a** shows the sacroiliac joint on plain radiographs, panel **b** shows the three-dimensional reconstruction dual-energy computed tomography (DECT) images, panel **c** shows the corresponding coronal (patient 2 and 4) or axial (patient 1 and 3) DECT images, and panel **d** shows the corresponding level of computed tomography (CT) images. A large quantity of MSU crystal deposition shown as *green* was found at the sacroiliac joint or the surrounding area in the DECT images. Four male patients (patient 1–4), aged 36, 44, 23, and 27 years old, had serum uric acid levels of 407 μmol/L, 370 μmol/L, 572 μmol/L, and 464 μmol/L, respectively. They were graded with a scale of 0, 0, II, III at the *left* sacroiliac joint and 0, I, II, III at the *right* sacroiliac joint, respectively, on plain radiographs

Examples of corresponding radiographic and DECT images of affected sacroiliac joints are shown in Fig. 2. Four male patients (patient 1–4), aged 36, 44, 23, and 27 years old, had serum uric acid levels of 407 μmol/L, 370 μmol/L, 572 μmol/L, and 464 μmol/L, respectively. As seen in Fig. 2a, four AxSpA patients were graded with a scale of 0, 0, II, III at the left sacroiliac joint and 0, I, II, III at the right sacroiliac joint, respectively, on plain radiographs. A large quantity of MSU crystal deposition was found at the sacroiliac joint or the surrounding area (Fig. 2b). The close relationship between MSU crystal deposition and radiographic structural damage (erosion, joint space narrowing, and new bone formation features) is shown at the sacroiliac joints of patients 2–4 (Fig. 2c-d).

## Discussion

The umbrella of AxSpA encompasses both nr-AxSpA and classic AS by ASAS [1, 2]. However, the range of diagnoses for AxSpA may cause difficulty among a large population with back pain, especially with sacroiliitis in gout or crystal deposition diseases [28]. First, the publication of classification criteria for AxSpA has produced some false-positive and false-negative cases, due to the way that the aforementioned diseases may mimic AxSpA in clinical manifestations. Second, the coexistence of AxSpA with other rheumatic diseases is common. For example, with an increasing mean age of AS onset and a decreasing mean age of gout onset [29, 30] AS coexisting with gout is more common [9]. Finally, at a certain stage of disease duration in AxSpA patients without the gout history, the deposition of MSU crystal may only be transient, which is hard to detect with traditional radiology methods, but has a positive impact on disease progression.

Fortunately, the recently developed DECT imaging method has provided higher sensitivity and specificity for MSU crystal deposition in patients with symptomatic gout and asymptomatic hyperuricaemia [19]. Therefore, a DECT finding of MSU crystal deposition has been included in the 2015 American College of Rheumatology and the European League Against Rheumatism (ACR/EULAR) gout classification criteria [31]. Although a subclinical MSU crystal deposition by DECT is insufficient for a diagnosis of gout, those depositions are, in fact, widespread in the body of patients or even normal individuals. A previous study reported that 20% of hyperuricaemic RA patients show DECT MSU crystal depositions, which are significantly associated with seronegativity [32]. Moreover, MSU crystal deposition was also observed in multiple joints and soft tissues of the body in patients with asymptomatic hyperuricaemia and was associated with increasing severity of coronary calcification [19, 20]. Indeed, we have found a few cases of MSU crystal deposition depicted in green with DECT at painful joints or skeleton regions not only in patients with asymptomatic hyperuricemia, but also in patients with iliac condensing ostitis, reactive arthritis, osteoarthritis, rheumatoid arthritis, and AxSpA in our clinical practice, when patients were considered for other causes of inflammatory activity. It may be meaningful that the MSU crystal deposited at those joints or skeleton regions. Those depositions may be involved in the process of inflammation and bone destruction in primary diseases. In this study we reported that a large amount of MSU crystal deposition detected with DECT was in pelvic, hip joint, and sacroiliac joint regions in AxSpA patients without gout. Those findings indicated that it might be easy to ignore these regions in patients with AxSpA when it coexists with MSU crystal deposition, which may play an important role in the occurrence or development of the disease.

As is commonly known, hyperuricaemia and MSU crystal deposition are the central risk factors for development of gout. Three stages of MSU crystal deposition have been proposed: reduced urate solubility, MSU nucleation, and MSU crystal growth [33]. But there is no inevitable corresponding relationship between hyperuricaemia, MSU crystal deposition, and gout. For example, a recent study has shown that imaging evidence of MSU crystal deposition appears only in 24% of asymptomatic patients [23]. In addition, our study also found MSU crystal deposits in AxSpA patients without gout history. Further analysis revealed that serum uric acid concentration was correlated with the volume of MSU crystal at the sacroiliac joints and the pelvis. Therefore, there are more details to be discovered in future research. On the one hand, it is still unclear why MSU crystal deposition forms in some joints, soft tissues, or skeleton regions of individuals with or without gout. There must be some factors in the microenvironment of the specific location that promote the deposition of MSU crystal. Examples are temperature, pH level, concentration of ions, and proteins all of which may also be influenced by interactions with each other [33]. On the other hand, it is currently not known whether the deposition of MSU crystal at different positions makes sense in the course of the disease. Indeed, some studies have indicated that silent deposition of MSU crystal in asymptomatic hyperuricemia patients is associated with more severe coronary calcification [20]. Interestingly, we also found that, in patients with AxSpA, the presence and volumes of MSU crystal at the sacroiliac joints were statistically different when grouped by radiographic grade, but not at the hip joint, pubic symphysis, or pelvis.

Previous studies have shown that smoking status, alcohol use, HLAB27 positivity, CRP, poor responsiveness to NSAIDs, and inflammation at magnetic resonance imaging (MRI) of the sacroiliac joint could predict the radiographic sacroiliitis progression in patients with AxSpA [34–36]. In this study, similar results were found, namely that disease duration, ESR, CRP, total back pain, PGA of disease activity, pain and swelling of peripheral arthritis, duration of morning stiffness, ASDAS scores, BASFI scores, and any use of biologic DMARDs were associated with the grade of sacroiliac joint damage in patients with AxSpA by bivariate analysis, apart from the presence and volumes of MSU crystal at the sacroiliac joint. Further, the ordinal logistic regression analysis also shows that the disease duration, BASFI scores, and volume of MSU crystal at the sacroiliac joint are associated with the structural joint damage of sacroiliac joints in patients with AxSpA when other potential factors have been adjusted.

Those results suggest that more attention should be paid to AxSpA patients with no response to anti-rheumatic therapy, especially in those with coexisting MSU crystal deposition. Additionally, it suggest that there might be some common pathogenesis or interacting mechanisms between AxSpA and MSU crystal deposition, which ultimately results in damage to the sacroiliac joint. On the one hand, the structural damage mentioned above occurs through alteration of physiological bone turnover with excessive osteoclast activation, which was abnormally regulated by the receptor activator of nuclear factor-κB (RANK), RANK ligand (RANKL), and the osteoprotegerin (OPG) signaling pathway [37, 38]. Although new bone formation evolving into ankylosis is an important feature of AxSpA, the process of inflammation and subsequent bone erosion also occurs synchronously [39]. Meanwhile, bone erosion was also recognized at some interface of MSU crystal deposition, which erodes the bone and cartilage to cause significant structural damage [23]. On the other hand, a large amount of common inflammatory and proinflammatory factors have been involved in the promotion of bone damage, such as tumour necrosis factor alpha, interleukin (IL)-1, IL-6 [40, 41]. Accordingly, our observation that volumes of MSU crystal at the sacroiliac joints are associated with the progress of radiographic grade at sacroiliac joints is consistent with the current understanding of the pathogenesis, but the exact interacting mechanism needs further investigation.

There are several limitations of this study. First, the disease category for AxSpA includes classic AS and nr-AxSpA [1, 2]. A subgroup analysis has not been performed, due to the limited sample size. Second, structural damage grading at the sacroiliac joint was estimated by plain radiograph, according to the modified New York criteria [22]. The radiological scoring methods with CT [42, 43], which has higher density resolution and repeatability, should be applied in the structural damage assessment. Third, this study is a cross-sectional analysis. Further prospective studies would be valuable to confirm the precise contribution of MSU crystal deposition to the structural damage of the sacroiliac joints in patients with AxSpA.

## Conclusions

In summary, the present study reveals that large quantities of MSU crystal deposition detected by DECT have been found in AxSpA patients without coexisting gout. In addition, it provides further evidence that the MSU crystal deposition at the sacroiliac joint in those patients has been associated with the progress of radiographic grade at sacroiliac joints, apart from AxSpA duration and BASFI score.

## Abbreviations

ACR/EULAR: American College of Rheumatology and the European League Against Rheumatism
AS: ankylosing spondylitis
ASAS: Assessment of SpondyloArthritis International Society
ASDAS: Ankylosing Spondylitis Disease Activity Score
AxSpA: Axial spondyloarthritis
BASFI: Bath Ankylosing Spondylitis Functional Index
CRP: C-reactive protein
DECT: Dual-energy computed tomography
DMARDs: Disease-modifying anti-rheumatic drugs
ESR: Erythrocyte sedimentation rate
MRI: Magnetic resonance imaging
MSU: Monosodium urate
NSAIDs: Non-steroidal anti-inflammatory drugs
PGA: Patient’s global assessment
SpA: Spondyloarthritis
VAS: visual analogue scale

## References

[1] Rudwaleit M, van der Heijde D, Landewe R, Listing J, Akkoc N, Brandt J, Braun J, Chou CT, Collantes-Estevez E, Dougados M, Huang F, Gu J, Khan MA, Kirazli Y, Maksymowych WP, Mielants H, Sorensen IJ, Ozgocmen S, Roussou E, Valle-Onate R, Weber U, Wei J, Sieper J (2009). The development of Assessment of SpondyloArthritis international Society classification criteria for axial spondyloarthritis (part II): validation and final selection. Ann Rheum Dis.

[2] Rudwaleit M, Landewe R, van der Heijde D, Listing J, Brandt J, Braun J, Burgos-Vargas R, Collantes-Estevez E, Davis J, Dijkmans B, Dougados M, Emery P, van der Horst-Bruinsma IE, Inman R, Khan MA, Leirisalo-Repo M, van der Linden S, Maksymowych WP, Mielants H, Olivieri I, Sturrock R, de Vlam K, Sieper J (2009). The development of Assessment of SpondyloArthritis international Society classification criteria for axial spondyloarthritis (part I): classification of paper patients by expert opinion including uncertainty appraisal. Ann Rheum Dis.

[3] Bakland G, Nossent HC (2013). Epidemiology of spondyloarthritis: A review. Curr Rheumatol Rep.

[4] Exarchou S, Lie E, Lindstrom U, Askling J, Forsblad-d’Elia H, Turesson C, Kristensen LE, Jacobsson LT (2016). Mortality in ankylosing spondylitis: results from a nationwide population-based study. Ann Rheum Dis.

[5] Smith JA (2015). Update on ankylosing spondylitis: current concepts in pathogenesis. Curr Allergy Asthma Rep.

[6] Itulescu TC, Alexandrescu C, Voinea LM (2014). Ocular involvement in spondylarthritis--new mechanisms, new therapies. Oftalmologia.

[7] Dundar U, Cevik H, Demirdal US, Toktas H (2014). Use of rituximab to treat a patient with coexistence of rheumatoid arthritis and ankylosing spondylitis: 18 months follow-up. Int J Rheum Dis.

[8] Borman P, Ayhan F, Okumus M (2011). Coexistence of rheumatoid arthritis and ankylosing spondylitis. Clin Rheumatol.

[9] Ho HH, Yu KH, Chen JY, Lin JL, Wu YJ, Luo SF, Liou LB (2007). Coexisting ankylosing spondylitis and gouty arthritis. Clin Rheumatol.

[10] Lourbopoulos A, Ioannidis P, Boura E, Antoniadis D, Karacostas D, Grigoriadis N (2013). Coexistence of multiple sclerosis and ankylosing spondylitis: report of two cases. Eur Neurol.

[11] Borman P, Tuncay F, Koybasi M, Ergun U, Inan L (2011). Coexistence of ankylosing spondylitis and multiple sclerosis. Acta Neurol Belg.

[12] Singh S, Sonkar GK, Singh U (2010). Coexistence of ankylosing spondylitis and systemic lupus erythematosus. J Chin Med Assoc.

[13] Gran JT (1985). An epidemiological survey of the signs and symptoms of ankylosing spondylitis. Clin Rheumatol.

[14] Abraham Z, Gluck Z (1997). Acute gout of the right sacroiliac joint. J Dermatol.

[15] Kwan BY, Osman S, Barra L (2013). Spinal gout in a young patient with involvement of thoracic, lumbar and sacroiliac regions. Joint Bone Spine.

[16] Konatalapalli RM, Demarco PJ, Jelinek JS, Murphey M, Gibson M, Jennings B, Weinstein A (2009). Gout in the axial skeleton. J Rheumatol.

[17] Glazebrook KN, Guimaraes LS, Murthy NS, Black DF, Bongartz T, Manek NJ, Leng S, Fletcher JG, McCollough CH (2011). Identification of intraarticular and periarticular uric acid crystals with dual-energy CT: initial evaluation. Radiology.

[18] Ogdie A, Taylor WJ, Weatherall M, Fransen J, Jansen TL, Neogi T, Schumacher HR, Dalbeth N (2015). Imaging modalities for the classification of gout: systematic literature review and meta-analysis. Ann Rheum Dis.

[19] Dalbeth N, House ME, Aati O, Tan P, Franklin C, Horne A, Gamble GD, Stamp LK, Doyle AJ, McQueen FM (2015). Urate crystal deposition in asymptomatic hyperuricaemia and symptomatic gout: a dual energy CT study. Ann Rheum Dis.

[20] Andres M, Quintanilla MA, Sivera F, Sanchez-Paya J, Pascual E, Vela P, Ruiz-Nodar JM (2016). Silent monosodium urate crystals deposits associate with severe coronary calcification in asymptomatic hyperuricemia: “an exploratory study”. Arthritis Rheumatol.

[21] Wallace SL, Robinson H, Masi AT, Decker JL, McCarty DJ, Yu TF (1977). Preliminary criteria for the classification of the acute arthritis of primary gout. Arthritis Rheum.

[22] van der Linden S, Valkenburg HA, Cats A (1984). Evaluation of diagnostic criteria for ankylosing spondylitis. A proposal for modification of the New York criteria. Arthritis Rheum.

[23] Dalbeth N, Aati O, Kalluru R, Gamble GD, Horne A, Doyle AJ, McQueen FM (2015). Relationship between structural joint damage and urate deposition in gout: a plain radiography and dual-energy CT study. Ann Rheum Dis.

[24] van der Heijde D, Lie E, Kvien TK, Sieper J, Van den Bosch F, Listing J, Braun J, Landewe R (2009). ASDAS, a highly discriminatory ASAS-endorsed disease activity score in patients with ankylosing spondylitis. Ann Rheum Dis.

[25] Machado P, Landewe R, Lie E, Kvien TK, Braun J, Baker D, van der Heijde D (2011). Ankylosing Spondylitis Disease Activity Score (ASDAS): defining cut-off values for disease activity states and improvement scores. Ann Rheum Dis.

[26] Calin A, Garrett S, Whitelock H, Kennedy LG, O’Hea J, Mallorie P, Jenkinson T (1994). A new approach to defining functional ability in ankylosing spondylitis: the development of the Bath Ankylosing Spondylitis Functional Index. J Rheumatol.

[27] Rajan A, Aati O, Kalluru R, Gamble GD, Horne A, Doyle AJ, McQueen FM, Dalbeth N (2013). Lack of change in urate deposition by dual-energy computed tomography among clinically stable patients with long-standing tophaceous gout: a prospective longitudinal study. Arthritis Res Ther.

[28] Braun J, Baraliakos X, Kiltz U, Heldmann F, Sieper J (2015). Classification and diagnosis of axial spondyloarthritis--what is the clinically relevant difference?. J Rheumatol.

[29] Yu KH, Luo SF (2003). Younger age of onset of gout in Taiwan. Rheumatology (Oxford).

[30] Will R, Calin A, Kirwan J (1992). Increasing age at presentation for patients with ankylosing spondylitis. Ann Rheum Dis.

[31] Neogi T, Jansen TLTA, Dalbeth N, Fransen J, Schumacher HR, Berendsen D, Brown M, Choi H, Edwards NL, Janssens HJEM, Lioté F, Naden RP, Nuki G, Ogdie A, Perez-Ruiz F, Saag K, Singh JA, Sundy JS, Tausche A, Vaquez-Mellado J, Yarows SA, Taylor WJ (2015). 2015 Gout classification criteria: an American College of Rheumatology/European League Against Rheumatism collaborative initiative. Ann Rheum Dis.

[32] Petsch C, Araujo EG, Englbrecht M, Bayat S, Cavallaro A, Hueber AJ, Lell M, Schett G, Manger B, Rech J (2016). Prevalence of monosodium urate deposits in a population of rheumatoid arthritis patients with hyperuricemia. Semin Arthritis Rheum.

[33] Chhana A, Lee G, Dalbeth N (2015). Factors influencing the crystallization of monosodium urate: a systematic literature review. BMC Musculoskelet Disord.

[34] Dougados M, Demattei C, van den Berg R, Hoang VV, Thevenin F, Reijnierse M, Loeuille D, Feydy A, Claudepierre P, van der Heijde D (2016). Rate and predisposing factors of sacroiliac radiographic progression after a 2 years follow-up period in recent onset spondyloarthritis. Arthritis Rheumatol.

[35] Poddubnyy D, Rudwaleit M, Haibel H, Listing J, Marker-Hermann E, Zeidler H, Braun J, Sieper J (2011). Rates and predictors of radiographic sacroiliitis progression over 2 years in patients with axial spondyloarthritis. Ann Rheum Dis.

[36] Blachier M, Canoui-Poitrine F, Dougados M, Lethuaut A, Fautrel B, Ferkal S, Le Corvoisier P, Farrenq V, Poulain C, Ghaleh B, Bastuji-Garin S, Claudepierre P (2013). Factors associated with radiographic lesions in early axial spondyloarthritis. Results from the DESIR cohort. Rheumatology (Oxford).

[37] Chhana A, Callon KE, Pool B, Naot D, Watson M, Gamble GD, McQueen FM, Cornish J, Dalbeth N (2011). Monosodium urate monohydrate crystals inhibit osteoblast viability and function: implications for development of bone erosion in gout. Ann Rheum Dis.

[38] McGonagle D, Wakefield RJ, Tan AL, D’Agostino MA, Toumi H, Hayashi K, Emery P, Benjamin M (2008). Distinct topography of erosion and new bone formation in achilles tendon enthesitis: implications for understanding the link between inflammation and bone formation in spondylarthritis. Arthritis Rheum.

[39] Jacques P, Lambrecht S, Verheugen E, Pauwels E, Kollias G, Armaka M, Verhoye M, Van der Linden A, Achten R, Lories RJ, Elewaut D (2014). Proof of concept: enthesitis and new bone formation in spondyloarthritis are driven by mechanical strain and stromal cells. Ann Rheum Dis.

[40] Punzi L, Scanu A, Ramonda R, Oliviero F (2012). Gout as autoinflammatory disease: new mechanisms for more appropriated treatment targets. Autoimmun Rev.

[41] Hreggvidsdottir HS, Noordenbos T, Baeten DL (2014). Inflammatory pathways in spondyloarthritis. Mol Immunol.

[42] Ozgocmen S, Ardicoglu O, Kaya A (2000). The relationship of clinical and laboratory measurements to two different radiological scoring methods in ankylosing spondylitis. J Back Musculoskelet Rehabil.

[43] Taylor HG, Wardle T, Beswick EJ, Dawes PT (1991). The relationship of clinical and laboratory measurements to radiological change in ankylosing spondylitis. Br J Rheumatol.

